# Using U‐Nets to Predict the Effects of Head Motion on Simulated Specific Absorption Rate for Ultra‐High Field Magnetic Resonance Imaging With Parallel Transmission

**DOI:** 10.1002/mrm.70363

**Published:** 2026-03-29

**Authors:** Katherine Anna Blanter, Alix Plumley, Alper Gungor, Shaihan Malik, Emre Kopanoglu

**Affiliations:** ^1^ Cardiff University Brain Research Imaging Centre (CUBRIC), School of Psychology Cardiff University Cardiff Glamorgan UK; ^2^ Department of Electrical and Electronics Engineering Bilkent University Ankara Ankara Türkiye; ^3^ Imaging Physics & Engineering Research Department and School of Biomedical Engineering and Imaging Sciences King's College London London Greater London UK

**Keywords:** deep learning, MRI safety, parallel transmit (pTx), specific absorption rate (SAR), ultrahigh field MRI

## Abstract

**Purpose:**

Ultrahigh‐field MRI requires careful management of the specific absorption rate (SAR), which is subject and subject‐position dependent. Within‐scan subject motion may exacerbate local SAR exposure, necessitating large safety margins to prevent SAR underestimation, which hampers imaging performance. This study proposes a U‐Net architecture to adapt safety calculations to motion as it happens, to facilitate high‐performance scanning without compromising safety.

**Methods:**

Electromagnetic simulations were performed for five body models at multiple positions with an 8‐channel parallel‐transmit coil. Q‐matrices were transformed into real‐valued SAR distributions—to train U‐Nets to estimate motion‐induced effects on local SAR—which were then mapped back to Q‐matrices. Separate U‐Nets were trained for different types of body motion (rightward/leftward/anterior/posterior/yaw), which were then cascaded to predict the effect of composite (off‐axis) and larger displacements on SAR. Finally, network‐estimated local SAR distributions were compared with ground truth after‐motion local SAR for realistic parallel‐transmit pulses.

**Results:**

Subject motion had a statistically significant effect on local SAR, but network‐estimated safety models recovered a faithful representation of the ground truth after‐motion local SAR. For the investigated parallel‐transmit pulses, the proposed approach reduced the safety margin from 2.14‐fold to 1.3‐fold and ensured more than 68% of the imaging performance could be realized while a safety model that includes all simulated subject positions would have limited scanning performance to as low as 21% of the maximum.

**Conclusions:**

The proposed position‐aware SAR calculation approach allows smaller safety margins, which has the potential to enable higher‐performance UHF‐MRI scanning without compromising safety for subjects who are unable to remain still.

## 1 Introduction

Ultra‐high field MRI (UHF‐MRI) offers improved SNR and contrast, allowing images to reveal finer anatomical details and subtler physiological effects [1]. However, the RF wavelength at UHF‐MRI is comparable to the volume being imaged [2], resulting in transmit‐field ($B_{1}^{+}$) inhomogeneity, leading to images containing diagnostically irrelevant intensity variations that may overshadow anatomical information [3]. Although adiabatic pulses can improve transmit‐field inhomogeneity effects, they have increased pulse duration and specific absorption rate (SAR) [4]. SAR is an intermediary parameter that relates the power absorbed in the tissue during MRI, to subsequent tissue heating. Therefore, it is used as one of the main safety parameters in MRI to ensure subject safety [5, 6, 7, 8]. $B_{1}^{+}$ inhomogeneities can also be addressed with parallel‐transmit arrays (pTx‐array) that provide simultaneous control of multiple transmit coils that are driven by tailored radiofrequency pulses [9, 10]. The benefits of pTx‐arrays are complicated by the possibility of inadvertently creating increased localized SAR (local SAR) in unpredictable locations due to constructive interference of electric fields (E‐fields) from the independently controlled pTx coils [11]. This has led to safety concerns, as local SAR limits can be reached with less power input than global SAR [12].

Concerns regarding increased local SAR in unpredictable locations are exacerbated with the prospect of unavoidable and unplanned subject motion and change in position within the coil after scan registration and calibration [13, 14, 15, 16]. For example, when evaluating all six degrees of motion at 7 T using pTx excitation, Kopanoglu et al. reported that peak local SAR can change location and increase by up to 3.1‐fold [13].

In practice, safety models used on MRI systems for real‐time safety calculations are unaware of actual subject position. In this case, safety models that are not sufficiently representative of possible subject positions may substantially underestimate SAR, with Kopanoglu showing up to 5.2‐fold SAR underestimation for both single‐channel and parallel‐transmit pulses, and for different slice orientations [14]. In contrast, safety models that contain all possible subject positions may prevent SAR underestimation, but are often unnecessarily cautious and impede the full potential of UHF‐MRI in the absence of motion. The same study showed that a mismatch between the position of the safety model being used and the actual subject position also influences SAR, with more than 4‐fold SAR overestimation observed for all parallel‐transmit pulses (with different numbers of spokes) and all target slices (from cerebellum to crown) [14]. Consequently, a safety model that can adapt to the subject in case of motion is desirable to ensure safety while maximizing scan performance.

Recent studies have utilized the computational efficiency offered by artificial intelligence towards realizing tailored safety models; Brink et al. [17] used convolutional neural networks to map T1‐weighted data to a local SAR distribution, Meliado et al. [18] used subject‐specific $B_{1}^{+}$ maps in a similar attempt, and Gokyar et al. [19] used $B_{1}^{+}$ and localizer‐like images for related ends. None of the studies considered the effect of motion on local SAR.

Subject motion also affects transmit coil sensitivities and therefore flip‐angle distributions ($B_{1}^{+}$‐maps) [20], and although $B_{1}^{+}$‐maps can be measured using MRI, in contrast to E‐fields which cannot, remeasuring $B_{1}^{+}$‐maps as subject motion happens is not practical. Therefore, Plumley et al. [21] proposed to use deep learning to predict the effect of motion on $B_{1}^{+}$‐maps. The study demonstrated that by training separate networks for a subset of motion types and amounts, and cascading these networks to approximate the actual motion, changes in $B_{1}^{+}$‐maps can be successfully estimated.

In this work, we use a similar approach to [21], but instead use deep learning to predict the effect of motion on simulated local SAR distributions. For this purpose, multiple realistic body models were simulated at multiple positions within a generic 8‐channel pTx‐array. Paired and labeled local SAR distributions preceding and following motion were used to train U‐Nets [22]. Deep learning estimated local SAR distributions were compared to ground‐truth counterparts to evaluate estimation quality. Finally, the estimated local SAR distributions were used to calculate local SAR for realistic pTx pulses and compared to ground‐truth local SAR.

## 2 Methods

### 2.1 Simulations

Simulations were carried out similarly to published work [13, 14, 21]. Briefly—electromagnetic (EM) simulations were conducted on five Virtual Population models (versions v. 3.1), Billie, Duke, Ella, Fats, and Glenn (IT'IS Foundation, Zurich, Switzerland), within a generic 8‐channel pTx‐array (coil‐element height/width/microstrip‐with: 110/40/3 mm, three capacitor slots, one feed port) tuned to 7 T (295 MHz) in Sim4Life version 7 (ZMT, Zurich, Switzerland). Similar to Plumley et al., Duke and Fats models were scaled to 90% of the original size, to avoid body and coil model intersection for some positions [21].

In the literature, translations and rotations over 20 mm/degrees have been reported in various patient groups [23, 24] as well as among elderly [25] and pediatric [26, 27] subjects [28]. Therefore, we repeated the simulations for one centered and 32 off‐center positions of the head (all combinations of 0, 2, 4, 5, 10, 20 mm rightward (R) and 0, 2, 4, 5, 10 mm posterior (P) shifts, as well as 5°, 10°, 15° rotations around the Yaw axis). As recommended in the literature, the neck and shoulders were included within the computational domain [29]. To ensure consistent tissue voxelization across all positions, the coil array was moved with respect to the body, identical to Ref. [13, 14, 21], also meaning that all field distributions were co‐registered. Three‐dimensional E‐field and current density distributions were mapped onto a predefined grid (size: 180 × 215 × 250 mm^3^, resolution: 1.5 × 1.5 × 1.8 mm^3^), and exported to MATLAB (The MathWorks, Natick, MA, USA), where they were converted to 10‐g averaged Q‐matrices (which contain the interaction of E‐fields from coil elements [30, 31]), using cubical volumes [5].

### 2.2 SAR Calculations

#### 2.2.1 Forward‐Mapping

The local SAR in voxel r can be calculated using:

(1) $$\mathrm{SAR}\left[r\right]=w^{H}\cdot Q\left[r\right]\cdot w$$

where w denotes pTx‐array channel coefficients, the superscript H denotes complex‐conjugate transpose, Q[r] denotes the complex‐valued N × N Q‐matrix that characterizes the interaction of the coil elements in voxel r, and N is the number of coil elements.

**FIGURE 1:**
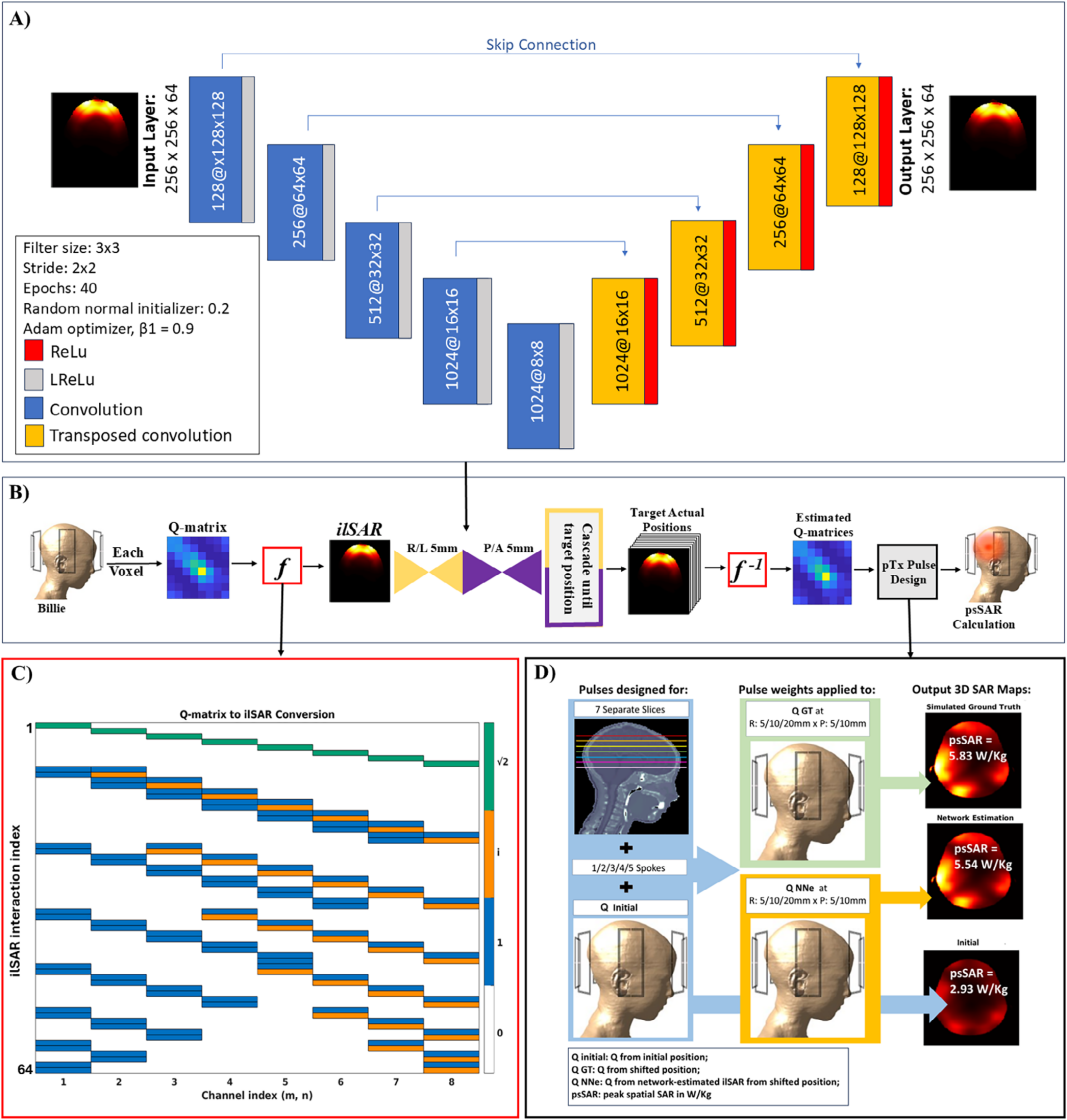
The neural network architecture is shown alongside the pipeline from body model simulation through local SAR calculation. (A) Neural network architecture. Five downstack layers are joined by skip connections (blue arrows) to four upstack layers. Activation functions were rectified linear unit (ReLu) for the deconvolution layers in the decoder path and leaky‐ReLu (LReLu) for the convolution layers in the encoder path. Hyperparameters are specified in the legend on the bottom left. (B) Q‐matrices extracted from electromagnetic simulations are fed through an algorithm to calculate real‐valued intermediate‐local SAR (ilSAR) distributions, which are then passed through neural networks to estimate ilSAR at an indicated shifted position. The estimated ilSAR distributions at the shifted position are remapped to Q‐matrices, which are used to calculate the realized local SAR distribution of a designed parallel‐transmit pulse and its peak spatial SAR value. (C) Illustration of the channel weights used for the forward mapping from Q‐matrices to ilSAR. Colors indicate channel combination weights. (D) pulses with 1‐, 2‐, 3‐, 4‐, or 5‐spokes were designed for seven different slices from cerebellum to crown for the initial position. Each pulse was evaluated with three sets of Q‐matrices; those at the initial position, actual (ground‐truth) Q‐matrices for the off‐center position, and estimated Q‐matrices at the off‐center position. Example case illustrated for a 2‐spokes pulse evaluated after an R20, P10 displacement.

To avoid estimating the complex‐valued entries of the Q‐matrices, these matrices can be characterized by a series of $N^{2}$ real‐valued projections, which we refer to as intermediate local SAR (ilSAR) distributions. This method was proposed by Zhu et al. [32] to enable estimation of Q‐matrices from (real‐valued) empirical power measurements (Figure 1C). For an N‐channel coil, the first N terms are single‐coil distributions given by:

(2) $$\text{ilSA}R_{m}^{c_{m}}\left[r\right]=w^{H}\cdot Q\left[r\right]\cdot w$$

where

(3) $$w\left[k\right]=\left\{\begin{array}{ll} c_{m} & ,k=m\\ 0 & ,\text{else} \end{array}\right.$$

The other N(N−1) ilSAR distributions are for when only two coils are on and the rest are off:

(4) $$\text{ilSA}R_{m,n}^{c_{m},c_{n}}\left[r\right]=w^{H}\cdot Q\left[r\right]\cdot w$$

where

(5) $$w\left[k\right]=\left\{\begin{array}{ll} c_{m} & ,k=m\\ c_{n}\;\text{or}\;\tilde{c_{n}} & ,k=n\\ 0 & ,\text{else} \end{array}\right.$$

and $c_{m}\;\text{and}\;c_{n}$ are coefficients of channels m and n. In this paper, we used $c_{m}=\sqrt{2}$ for single channel ilSAR and two pairs of channel coefficients $\left(c_{m},c_{n}\right)=\left(1,1\right)$ and $\left(c_{m},\tilde{c_{n}}\right)=\left(1,i\right)$ for each two‐coil interaction.

#### 2.2.2 Reverse‐Mapping

The network estimated ilSAR distributions were inverse mapped to estimated Q‐matrices, (denoted by Qm,nestim) using the general solution given in Equation (A1) in the Appendix, which reduces to Equation (6) for the coefficients used in this study:

(6) Qm,nestim=ilSARmcmestim2,m=n12ilSARm,ncm,cnestim+−i2ilSARm,ncm,cn˜+estim−1+i4ilSARmcm+−1+i4ilSARncnestimestim,m<n12ilSARm,ncm,cnestim+i2ilSARm,ncm,cn˜+estim−1−i4ilSARmcm+−1−i4ilSARncnestimestim,m>n

where subscripts and superscripts denote channels and channel coefficients, respectively.

### 2.3 Dataset

To prepare the data for processing via neural networks, ilSAR data were normalized by the overall maximum value across all body models, positions and (axial) slices, and interpolated to 256 × 256 in‐slice resolution. Data were processed slice‐by‐slice, with coil combinations of the pTx‐array concatenated in the third dimension, yielding data size 256 × 256 × 64 for training neural networks per slice. The number of axial slices that contained tissue data varied between 126 and 136 across body models due to different body model sizes.

Because SAR/Q‐matrix/ilSAR distributions show similarity in adjacent slices, splitting data into training, validation, and testing datasets slice‐wise can introduce crosstalk between the datasets and produce misleading results. Therefore, datasets were split at the body model level, with Ella, Fats, and Glenn forming the training, Duke the validation, and Billie the testing datasets. This approach ensures that the testing dataset is unseen by the networks during training.

To create the training datasets, data from before and after motion were paired for all combinations of positions that yielded the same relative displacement. For example, for rightward 5 mm motion, pairs of positions included: R0→R5; R5→R10; R5, P5→R10, P5; etc. where R5 and P5 denote 5 mm rightward and posterior displacements, respectively. The number of pairs of position for rightward/posterior/yaw was 10/12/3. During initial development, we discovered that the smooth and low‐frequency spatial variations of ilSAR distributions made U‐Nets susceptible to over‐fitting. To reduce over‐fitting, we only included every third slice in the training dataset (and discarded the rest), yielding 1560 cases for posterior, 1300 cases for rightward motion, and 402 cases for yaw rotation.

For validation and testing, we used all slices (Duke: 135, Billie:136). For validation, we used only center‐out position pairs of exact displacement (i.e., R0→R5 for rightward 5 mm). To test network performance outside the amount of displacement networks were trained for (e.g., R5), we paired all simulated positions with the centered position as reference (e.g., R0→R20), enabling testing of the networks with larger displacements (e.g., R20 mm) by cascading the networks as necessary.

### 2.4 Investigations

#### 2.4.1 Rightward‐Posterior

Two separate, architecturally identical neural networks were trained on R5 and P5 displacements.

#### 2.4.2 Off‐Axis Displacements and Beyond 5 mm

It is impractical to train separate networks for all motion types and amounts. Therefore, we cascaded networks to investigate evaluation performance for larger displacements and off‐axis displacements, similar to Ref. [21] (Figure 1B). To cascade, the output of one evaluation network provided the input for a subsequent network until the target evaluation was reached. For example, four copies of an R5 network needed to be cascaded with two P5 networks to estimate the effect of R20, P10 motion, which will be denoted in future sections and tables as RRRR5 PP5.

#### 2.4.3 Leftward‐Anterior

We also investigated translations paired in the opposite directions: leftward and anterior. For this purpose, we used the same dataset but with reversed pairing, that is, R5 labeled as the reference position for R0 (i.e., R5→R0), emulating 5 mm leftward movement (L5) (similarly for posterior and anterior (A)).

#### 2.4.4 Yaw

The suitability of the proposed method to rotations was investigated using Yaw 5° (Y5) networks with an identical cascading mechanism.

We did not include superior–inferior displacement, pitch, or roll rotations in this proof‐of‐principle study because previous work [13] determined that local SAR varies most with translation in the axial plane.

#### 2.4.5 Cascading Effect on Error

We investigated the effect of cascading on estimation performance via four scenarios:

- Estimation error floor: Different combinations of networks were cascaded that summed up to zero displacement (i.e., back to the initial position), such as R5, L5.
- Cascading order: Different cascading orders that sum to the same total displacement were compared, such as RR5, PP5 and R5, P5, R5, P5 for R10, P10 displacement.
- Number of cascades: Cascading P5 twice for P10 displacement was compared with a network directly trained for P10 motion that did not require any cascading. Because there was more data available for training the P5 network (P0→P5 and P5→P10) than for the P10 network (only P0→P10), we also trained an alternative P5 network with a smaller dataset (940 pairs) similar in size to that of the P10 network (782 pairs), dubbed here P5(smaller dataset).
- Off‐grid displacement: The efficiency of estimations was investigated for when there is a positional mismatch, such as when motion tracking information is imperfect or when cascading the networks cannot reach the actual subject position (i.e., a displacement which the network was not trained on) by comparing the estimations of the R5 network to evaluate R4 motion.

#### 2.4.6 Fourfold Cross‐Validation of Body Models

We tested the approach in a fourfold cross validation investigation similar to [17, 18] by shuffling the body models in the training/validation/testing datasets, for P5 and P10 motion. In addition to the previous testing performed on a female pre‐adolescent body model (Billie), these investigations tested the networks on obese adult male (Fats), elderly adult male (Glenn), and adult female (Ella). Body model BMIs ranged between 15.3 and 36 (Figure 4A). The configurations are denoted by initials of body model names grouped in order of training (three models)–validation–testing: EBG‐D‐F, FED‐B‐G, FDB‐G‐E whereas the other investigations in this manuscript are for EFG‐D‐B (Ella/Fats/Glenn‐Duke‐Billie).

#### 2.4.7 Dataset Preparation Method

We compared our dataset‐preparation method (split by body model, referred from here onward as leave‐one‐out [LOO]) to the slice‐interleaved (SI) approach from Ref. [19], for which we split the whole dataset as 2/3 training, 1/6 validation and 1/6 testing. We trained P5 network with both LOO and SI datasets, then compared P5 and P10 (P5 cascaded twice) results.

### 2.5 Network

The neural network (Figure 1A), implemented in TensorFlow [33] version 2.4.1, contained five downstack layers joined by skip connections to four upstack layers. The activation functions were rectified linear unit (ReLu) for the deconvolution layers in the decoder path and leaky‐ReLu (LReLu) for the convolution layers in the encoder path. The first three deconvolution layers were joined by dropout layers (dropout rate, 0.5). Other network parameters are indicated in Figure 1.

We reached optimal training performance at 40 epochs. The networks took ˜4 h to train on a NVIDIA (Santa Clara, CA, USA) DGX v‐100 GPU.

### 2.6 Post‐Processing

Once the networks were trained separately for each motion type (A5/P5/L5/R5/Y5), they were evaluated using the testing dataset from the Billie model (Figure 1B). Estimated ilSAR distributions were exported to MATLAB and masked as a precaution to remove negligible spurious numerical artifacts in the background. To remove estimation noise in regions where initial field values are minute, distributions were smoothed using a Gaussian kernel, after which larger magnitudes (larger than 1% of the maximum) were restored to their original values.

Network prediction quality was quantified at each position using normalized root‐mean‐squared‐error (nRMSE), between ground‐truth simulations and network‐estimated ilSAR:

(7) nRMSE=100×1Nr∑rNrilSARm,ncm,cnestim−ilSARm,ncm,cnGT21Nr∑rNrilSARm,ncm,cnGT2,

where ilSARm,ncm,cnestim and ilSARm,ncm,cnGT are the estimated and ground‐truth ilSAR distributions for a given channel combination, r indexes the pixels within a slice and $N_{r}$ is the number of pixels within a slice. The effect of motion was quantified by calculating the nRMSE between ground‐truth simulations and initial position local ilSAR.

### 2.7 Parallel Transmit Pulses

Slice‐selective pTx pulses with 1/2/3/4/5‐spokes were designed to create homogeneous in‐slice excitation at the centered position [20, 34] (Figure 1D), for seven different slices from cerebellum to crown, with 1.8 mm slice separation. During optimization, the channel‐by‐channel RF power was controlled using Tikhonov regularization. Three‐dimensional 10‐g averaged local (equivalently, spatial) SAR distributions were calculated using centered, ground‐truth off‐center and estimated off‐center Q‐matrices to investigate the effect of motion on SAR, similar to the literature [13]. In other words, we assume knowledge of the ideal safety model and exclude the mismatch between the safety model and the actual body to focus on the effect of motion and the correction our models provide. Network‐estimated 3D SAR distributions were passed through a 3D binomial filter for smoothing, where the element in position [i,j,k] is given by: $I_{3D}\left[i,j,k\right]=I_{1D}\left[i\right]\times I_{1D}\left[j\right]\times I_{1D}\left[k\right]$ where $I_{1D}=\left[1/4,1/2,1/4\right]$. Peak spatial 10‐g averaged SAR (psSAR) values were also calculated.

We hypothesized that subject motion has a substantial effect on psSAR which can be corrected using deep learning. We tested the hypothesis using a two‐sample Kolmogorov–Smirnov test [35, 36] with a significance level *p* = 0.05 on psSAR values from 35 pulses that were designed for the centered position and evaluated at 11 other positions (combinations of *R* = 5, 10, 15 and *p* = 5, 10 displacements), yielding 385 samples.

## 3 Results

### 3.1 Network‐Estimation

#### 3.1.1 Rightward‐Posterior

**FIGURE 2:**
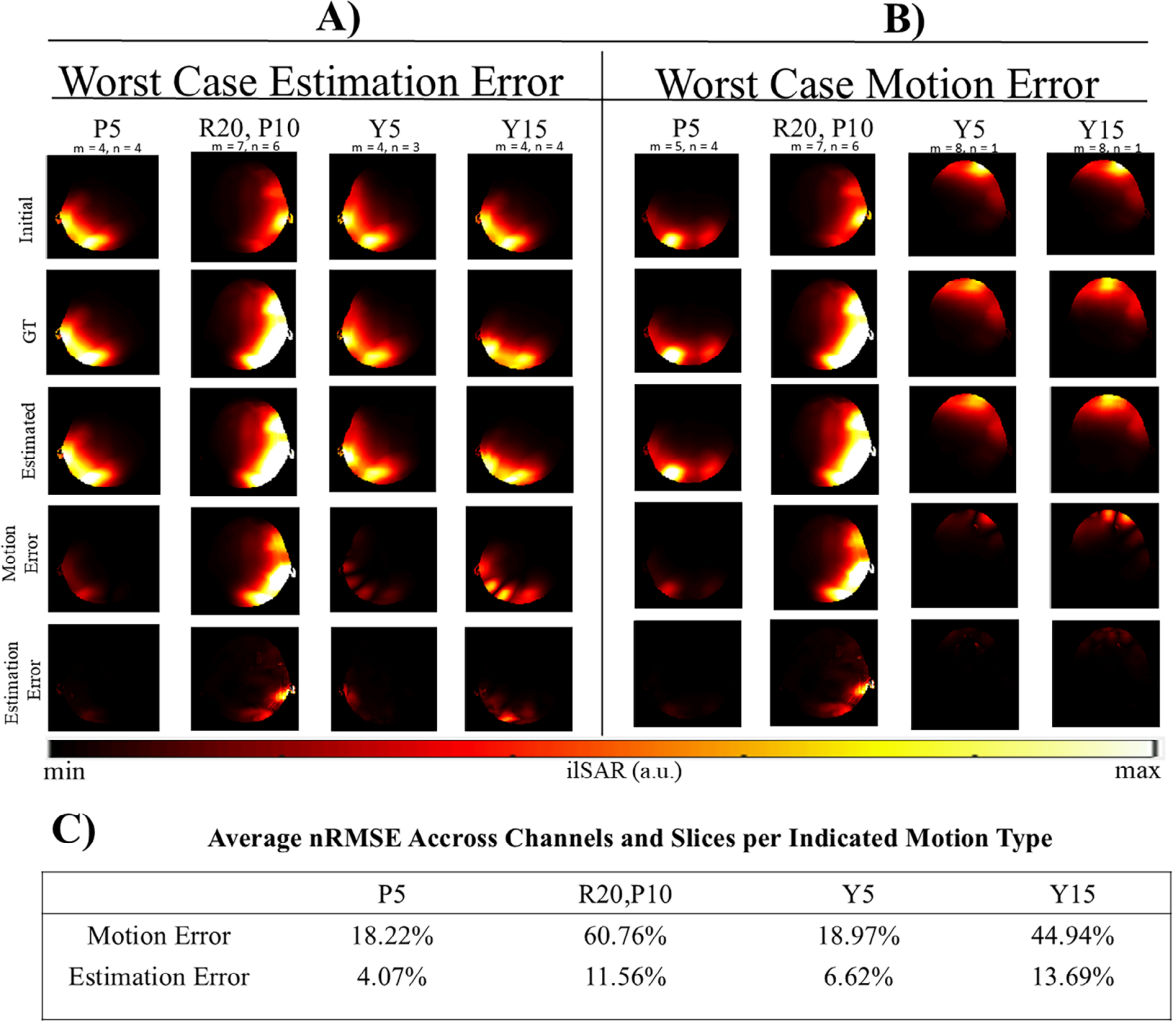
Normalized ilSAR distributions before motion, after motion (ground‐truth), and after estimation are shown for four motion states (P5; R20, P10; Y5; Y15). Section (A) of the figure shows the ilSAR channel combinations that led to the largest network‐estimation error (|network‐estimated‐ ground‐truth|), while Section (B) shows the ilSAR combinations that led to the worst‐case motion error (|initial‐ ground‐truth|). Each column contains maximum intensity projections (MIP) of 3D distributions onto the axial plane. Channel indices m and n indicate which two‐channel interaction is shown in each column. In order, the top three rows show the MIPs at the “Initial” position, the ground‐truth (“GT”) and network‐estimated (“Estimated”) at the off‐center positions. (C) The two bottom rows show the motion‐induced and network‐estimation errors (“Motion Error”: Row 2—row 1 and “Estimation Error”: Row 2—row 3, respectively). Color ranges are consistent within each column with corresponding first row.

Figure 2 shows example ilSAR distributions for four off‐center positions. For each position, two channel combinations are presented, one that yields the worst estimation error and one that yields the worst motion error. In all cases, including those selected to show the worst estimation performance, the networks visibly reduce motion‐induced SAR calculation errors.

For a single body model containing 136 slices and 64 channels, SAR correction with the networks took an average of 16 ± 0.002 ms per slice. This is the value for network runtime excluding network loading time, since the authors envision that in practice, the network weights will be preloaded.

**FIGURE 3:**
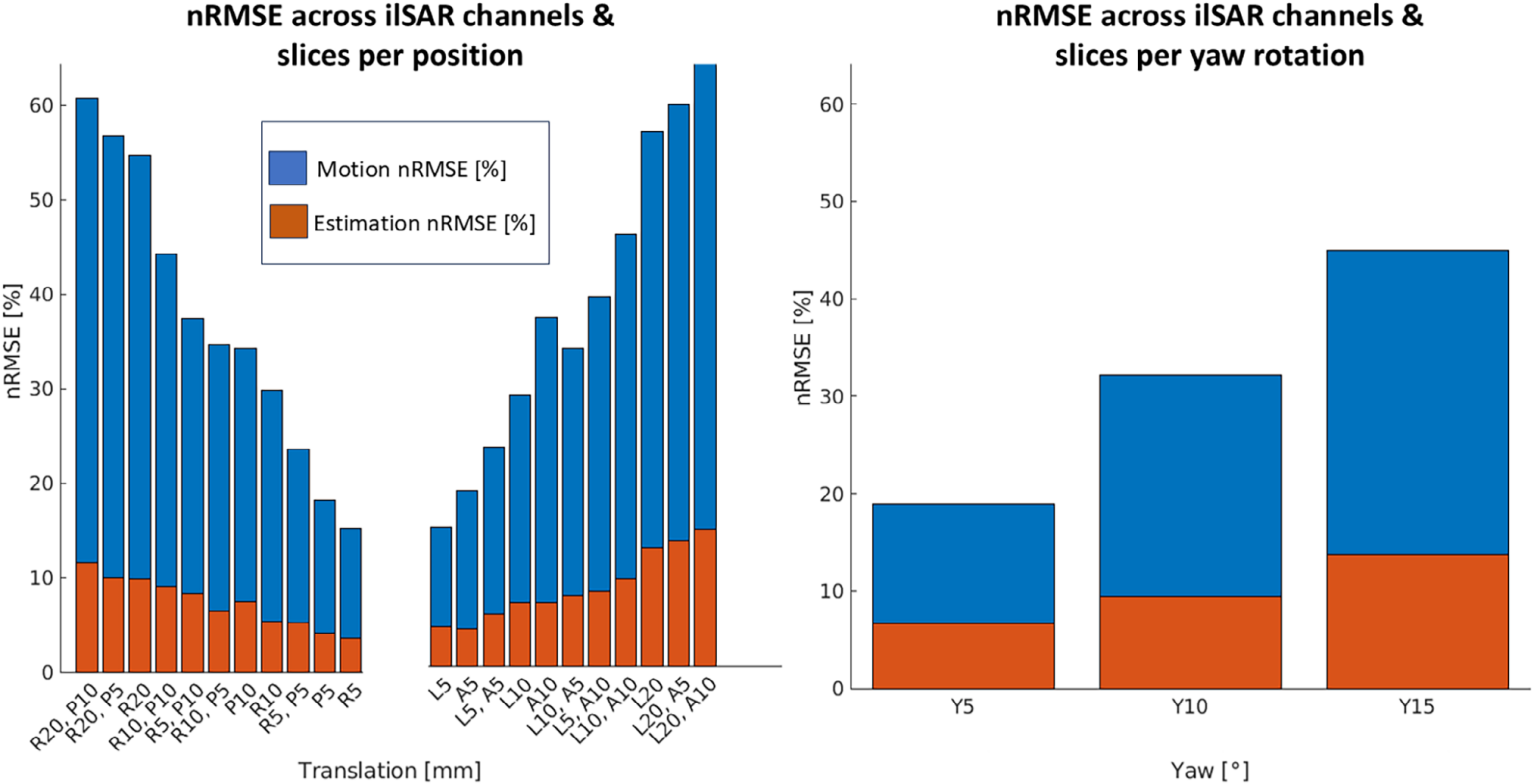
Motion‐induced and network‐estimated nRMSE (%) values, averaged across slices and channels, are shown for each degree of motion. R: Rightward, L: Leftward, A: Anterior, P: Posterior, Y: Yaw. Values in the left panel are displayed in descending(R)/ascending(L) order of radial displacement from the center of the RF coil. For equivalent radial distances, leftward and rightward increases are positioned closer to the center than anterior and posterior; i.e., L10, A5 closer to center than L5, A10.

For all cases investigated, networks reduced motion error considerably. Figure 3 shows nRMSEs averaged across all 136 slices and 64 channels for each motion case. Network estimations reduced the motion‐induced ilSAR from a worst‐case of 67.2%–14.5%, and an average of 37.9% across positions to 7.8% for axial translations. For yaw rotations, network estimation reduced ilSAR nRMSE from a worst‐case of 44.9%–13.7%, and an average of 32.0%–9.9%. A paired *t*‐test showed that all reductions were statistically significant (*p* = 0.01).

Figure 3 shows that the motion‐induced error increases monotonically with increasing displacement (translational or rotational) from the initial position, with the only exception being the error for A10 being larger than L10, A5. Furthermore, anterior and posterior displacements yielded more error than rightward and leftward displacements of equivalent distances; that is, the error for A10, L5 was larger than A5, L10.

#### 3.1.2 Cascading

The results of the four cascading scenarios are reported in Table 1 and discussed in detail below.

**TABLE 1:** Alternative cascading strategy results.

Cascade type	Estimated nRMSE	Motion nRMSE
**Estimation error floor: Cascade to 0 (no motion)**		
R5, L5	3.18%	N/A
P5, A5	3.19%	
RPLA5	5.56%	
RR5, LL5	4.78%	
R5, LL5, R5	4.91%	
AA5, PP5	4.55%	
RR5, PP5, LL5, AA5	18.55%	
**Cascading order: cascade to R10, P10 mm**		
RR5, PP5	9.06%	44.25%
PP5, RR5	9.86%	
R5, PP5, R5	9.36%	
R5, P5, R5, P5	9.36%	
PP5, AA5, PP5, RR5, LL5, RR5	24.96%	
**Number of Cascades: Cascade to P10 mm**		
PP5	7.42%	34.2%
PP5 (smaller dataset)	9.53%	
P10 trained directly	10.52%	
**Off‐grid Displacement: R4 mm**		
R5	3.83%	12.20%

*Note*: Network‐estimation (network estimation vs. ground truth) and motion‐induced (initial position vs. ground truth) error (nRMSE [%]: normalized root‐mean‐squared‐error) from alternative cascading methods. A: anterior motion, L: leftward, P: posterior, R: rightward, Numerical values indicate displacement value in mm.

##### 3.1.2.1 Estimation Error Floor

When networks were cascaded to yield zero total displacement, the error compared to the initial data increased with the number of networks cascaded; two‐network cascades, RL5 and PA5, yielded 3.19% and 3.18% error, respectively, while four‐network cascades (RPLA5; RR5, LL5; R5, LL5, R5; AA5, PP5) yielded a mean of 4.95% ± 0.43% error. The error for the eight‐network cascade (RR5, PP5, LL5, AA5) was 18.55%. Across all cases, error introduced by a network was calculated as 1.50% ± 0.41%.

##### 3.1.2.2 Cascading Order

For the tested cases (RR5, PP5; PP5, RR5; R5, PP5, R5; R5, P5, R5, P5), the influence of cascading order on estimation performance was small, with nRMSE values ranging between 9.06% and 9.86% with a sample standard deviation of 0.33%.

When 12 networks were cascaded instead of four by inserting eight additional networks that internally add up to zero displacement (PP5, AA5, PP5, RR5, LL5, RR5), error increased by 15.1%–24.96%, which is in alignment with the expected 1.50% ± 0.41% increase per network from the previous investigation. To contextualize the estimation error of 24.96%, the motion induced error for R10, P10 was notably higher at 44.25%.

##### 3.1.2.3 Number of Cascades

Next, the effect of training separate networks for larger movements was compared with cascading networks trained for smaller movements, using the example case P10 versus PP5. The default P5 network trained with more data provided the best performance with an nRMSE of 7.42%, which increased to 9.53% for the P5 (smaller dataset) network and to 10.52% error for the purpose‐trained P10 network. For comparison with the different cascade scenarios investigated, the motion‐induced nRMSE was 34.20%.

##### 3.1.2.4 Off‐Grid Displacement

Finally, we investigated when the actual motion does not fall on the grid for which networks were trained for, by evaluating the performance of the R5 network in estimating the effect of rightward 4 mm motion (R4), which was outside the training conditions—i.e., comparing 5 mm estimated maps with 4 mm actual displacement maps. While motion‐induced nRMSE from the R4 displacement was 12.20%, network‐estimation reduced the nRMSE to just 3.83%. To compare, the motion‐induced nRMSE was 15.21% for R5 mm motion and the estimation error was 3.61%. The network‐estimation error for R4 mm is higher than that of R5 mm despite the motion‐induced error being lower due to smaller displacement. This is expected as R5 mm is on the grid of positions the network was trained for while R4 mm was outside the training conditions. Nevertheless, the network was still able to reduce the motion‐induced error considerably.

#### 3.1.3 Fourfold Cross Validation

**FIGURE 4:**
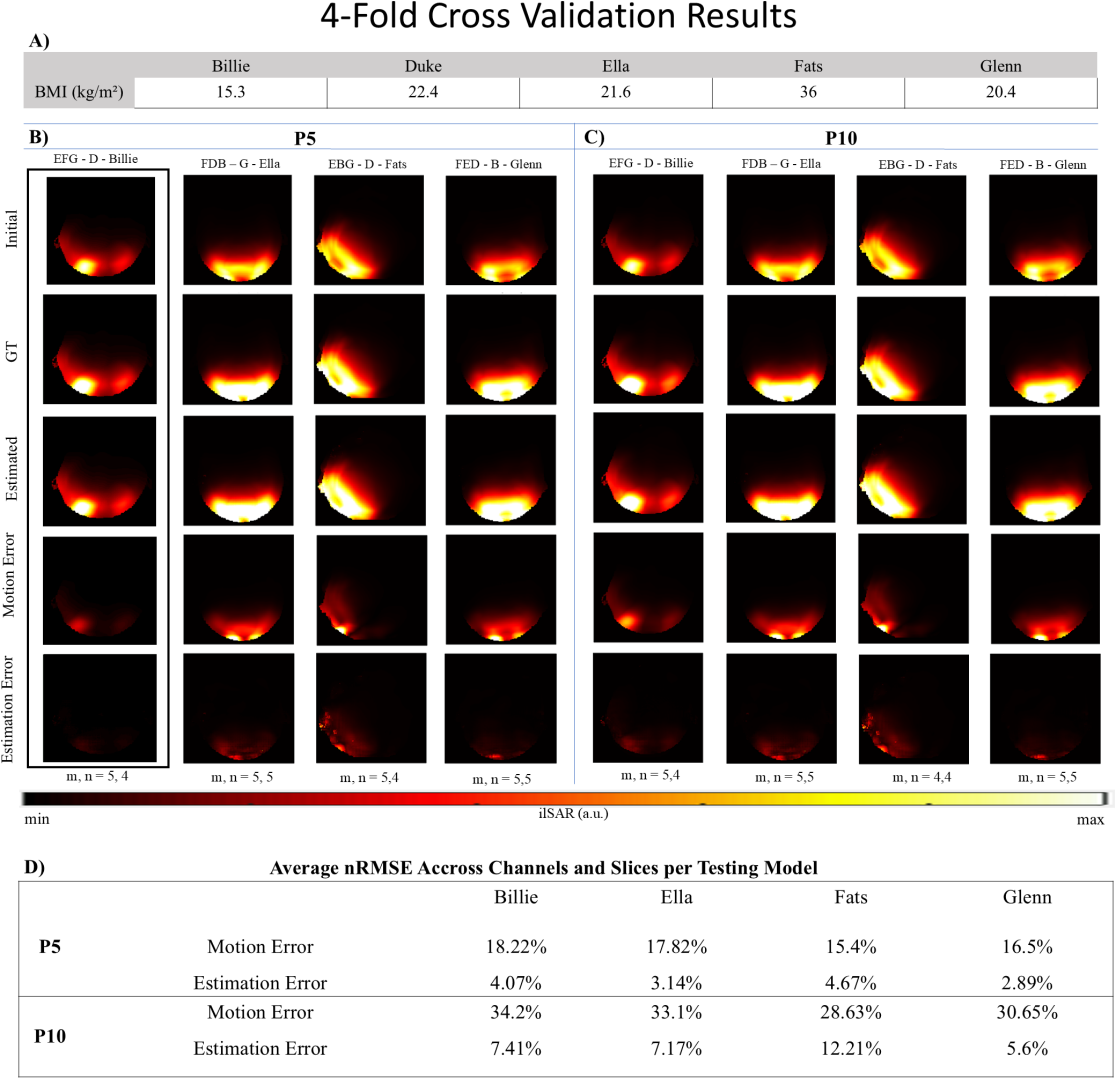
Cross‐validation of proposed method on alternative body models for channel combinations with worst‐case motion‐induced error. The letters F, D, G, B, E indicate Fats, Duke, Glenn, Billie, Ella, and the grouping (e.g., EFG‐D‐B) indicates the three models used for training (e.g., Ella, Fats, Glenn), followed by the model used for validation (e.g., Duke) and finally the testing model (e.g., Billie). (A) BMI values in kg/m^2^ per body model. (B) Each column shows P5 network testing results from the body models indicated at the top. Each row, in order, is Initial (before motion), ground truth (GT; after motion), Estimated (network‐estimation of ilSAR distribution after motion), motion‐induced error (Motion Error; |Initial—GT|), and network estimation error (Estimation Error; |Estimation—GT|). Numerical values on the error maps indicate the in‐slice nRMSE averaged across slices and channels. The first column (highlighted by the rectangle) is repeated from Figure 2 for easier reference. (C) Similar to panel (B) but for P10motion. (D) Average nRMSE (%) accross channels and slices per testing model.

For both P5 and P10 motion, the networks reliably reduced the error in ilSAR due to motion across all investigated motion types and body model configurations (Figure 4B,C). For P5 motion (Figure 4B), motion‐induced nRMSE averaged across slices and channels varied between 15.4% and 18.2% across the testing models, whereas the network estimations reliably reduced the error to between 2.9% and 4.7%. For P10 displacement (Figure 4C), motion‐induced error was between 28.3% and 34.2%. For the three testing models with similar BMI, the error was between 5.6% and 7.4% whereas for the highest‐BMI model it was 12.2%.

#### 3.1.4 Dataset Preparation Method

Because the data are processed in different ways, the networks trained/validated/tested using LOO and SI data‐processing have different testing data, with motion‐induced error at 18.2% (LOO) and 17.1% (SI) for P5, and 34.2% (LOO) and 31.8% (SI) for P10. The P5 network trained/validated/tested on different body models (LOO) reduced motion‐induced error to 4.07% (P5) and 7.4% (P10, 2× cascades), averaged across slices and channels, resulting in relative error reductions of 77.7% and 78.3%, respectively, whereas the network that used SI data reduced error to 3.1% and 6.1%, yielding 82.1% and 80.8% relative error reduction, respectively (Table 2).

**TABLE 2:** Alternative dataset preparation results (SI vs. LOO).

	P5 mm	P10 mm
SI Estim	3.06%	6.1%
SI motion	17.05%	31.79%
LOO Estim	4.07%	7.42%
LOO motion	18.22%	34.2%
SI improvement	82.05%	80.81%
LOO improvement	77.66%	78.3%

*Note*: Comparison of (nRMSE [%]: normalized root‐mean‐squared‐error) resulting from the slice interleaving (SI) and leave‐one‐out (LOO) data preparation methods for P5 and P10 (2 × P5 cascaded, i.e., PP5) networks. “Estim” is network estimation error (network estimation vs. ground truth), while “motion” indicates motion‐induced error (initial position vs. ground truth). P: posterior displacement. Numerical values indicate displacement value in mm.

### 3.2 Parallel‐Transmit Pulses

**FIGURE 5:**
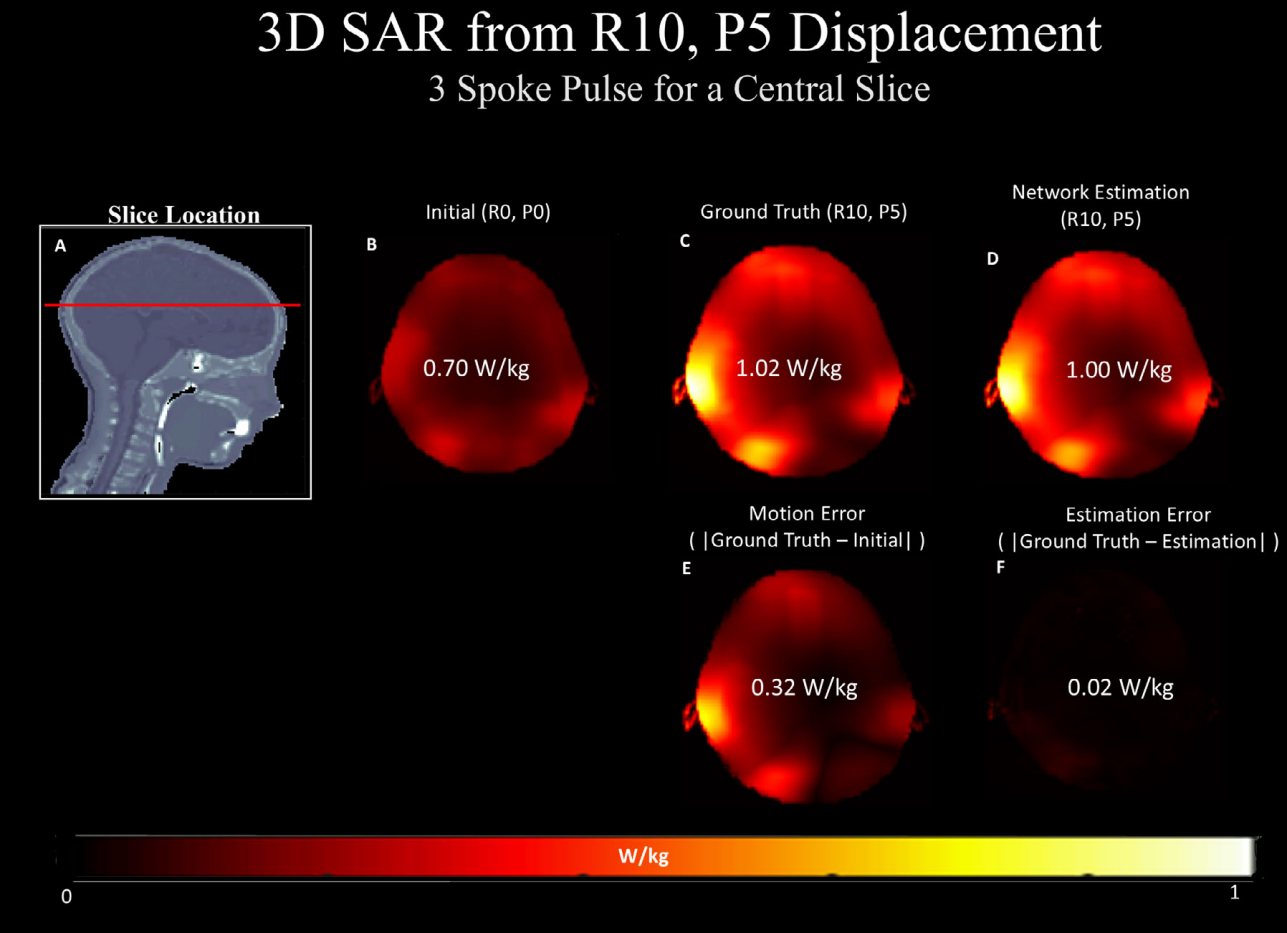
The maximum intensity projection (along the z‐axis) of the 3D SAR distributions for R10, P5 motion for a typical realistic 3‐spoke parallel‐transmit pulse designed for the central slice. (A) *T*he position of the slice the pulse was designed for. (B) Simulated SAR distribution at the initial position, that is, before motion. (C) Simulated ground‐truth SAR distribution *after motion* at R10, P5. (D) Network‐estimated SAR distribution after motion at R10, P5. (E) Motion‐induced error compared to the ground‐truth (i.e., |Ground Truth—Initial|). (F) Network‐estimation error compared to the ground‐truth (i.e., |Ground Truth—Network Estimated|). All panels share the same color scale range.

For each designed pTx pulse, three‐dimensional and peak 10‐g averaged local SAR were calculated using centered (initial), ground‐truth off‐center, and network‐estimated off‐center Q‐matrices. Figure 5 compares 10‐g averaged local SAR distributions for an example case. Calculating the after‐motion SAR distribution using the centered Q‐matrices highly underestimates SAR, whereas the network‐estimated safety model provides a visually nearly identical estimation of the ground‐truth SAR distribution. The psSAR values were 1.02 W/kg for ground‐truth off‐center SAR, 0.70 W/kg calculated using the centered Q‐matrices and 1.00 W/kg with the network‐estimated Q‐matrices, yielding only 2% estimation error in this instance.

**FIGURE 6:**
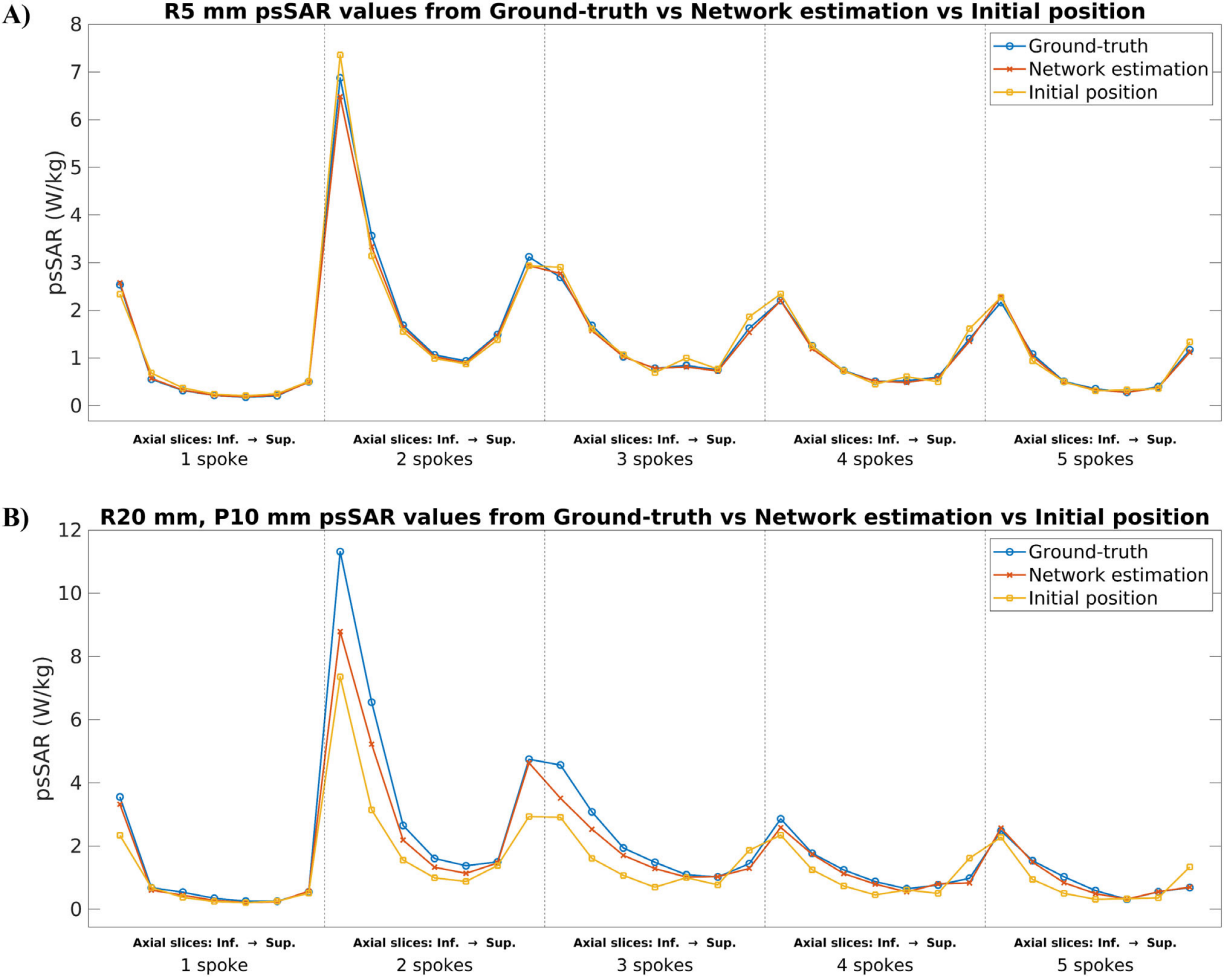
Comparison of ground‐truth, network‐estimation, and initial‐position psSAR values for R5 and R20, P10 motion, displayed for each spoke and slice combination. (A) R5 motion. (B) R20, P10 motion. While smaller displacements such as R5 showed minimal need for improvement, it is evident that the proposed deep learning method is beneficial for greater displacements such as R20, P10.

Figure 6 shows that in alignment with previous literature [13], motion‐induced psSAR error increases for larger displacements. The network estimated psSAR values closely follow ground‐truth psSAR for smaller displacements (Figure 6A), while considerably reducing motion‐induced calculation errors for larger displacements (Figure 6B). The worst‐case motion‐induced error was reduced from 53.23% to 13.50% by the networks, whereas the average motion‐induced nRMSE across all pulses was reduced from 35.9% to 13.5%. The greatest reduction in error occurred at an inferior slice with 5 spokes, where a 16.90% SAR underestimation was reduced to 3.38% by networks, indicating an 80% relative reduction in SAR estimation error.

**FIGURE 7:**
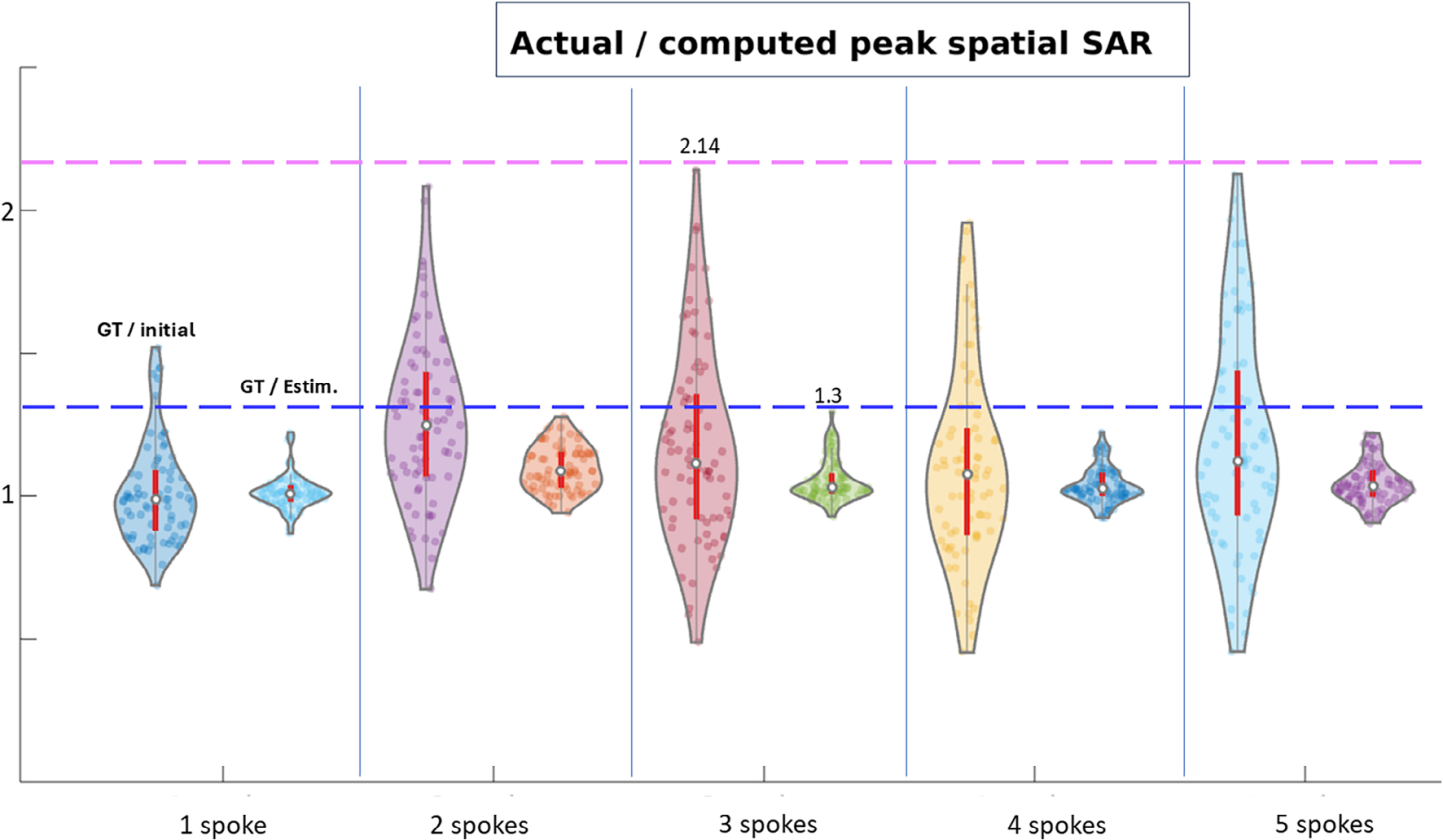
SAR underestimation due to motion and network‐estimations is shown, separately for different pulse types and collated across target slices and evaluated positions. Within each panel, left: Ground‐truth (motion) psSAR/initial (no motion) psSAR, and right: Ground‐truth (motion) psSAR/network‐estimated (motion) psSAR. In the worst SAR underestimation case, actual psSAR increased by 2.14‐fold due to motion (pink dashed line), whereas the networks reduced the estimation error to 1.3‐fold (blue dashed line). Red boxplots depict inter‐quartile range.

Figure 7 compares the motion‐induced and network‐estimated error in psSAR for all 35 pulses designed evaluated at all positions. For all pulses, the network estimations represent the actual SAR faithfully, indicated by the narrow range of values that demonstrate reduced underestimation and reduced overestimation.

**FIGURE 8:**
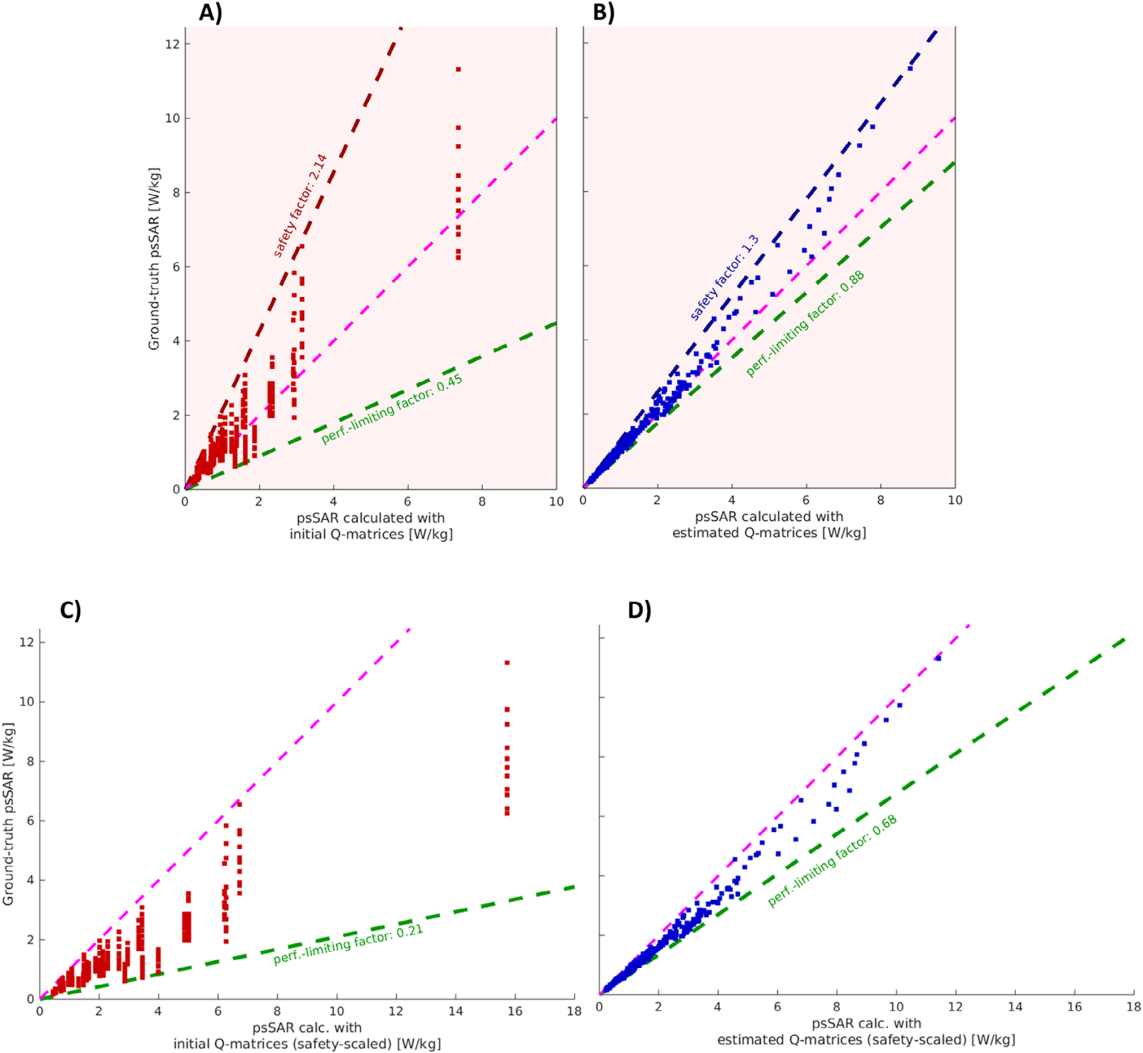
Initial and network‐estimated psSAR values are plotted against ground‐truth SAR. (A) initial psSAR vs. motion‐induced (ground‐truth) psSAR. (B) network‐estimated psSAR vs motion‐induced psSAR. Panels (A) and (B) show the raw SAR calculations. Panels (C) and (D) show the scaled calculations when the worst‐case underestimation factor is applied as a corrective safety margin to ensure SAR is never underestimated. The safety margin limits imaging performance, and the worst‐case SAR overestimation is indicated as “perf.‐limiting factor” on the figures (green dashed line). For the initial model in panel (C), performance was limited to 21% of the maximum, whereas the limitation was 68% when the networks were used to estimate SAR. Pink dashed line: Identity line.

Figure 8A,B, which collate the pulse‐wise results in Figure 7 into a single comparison, show that the initial safety model fails to capture the range of SAR values after motion, which are successfully recovered by the network‐estimated safety model. Across all cases, psSAR was underestimated by up to 2.14‐fold by the initial SAR distribution whereas the worst‐case underestimation was only 1.3‐fold for the network‐estimated safety model. These results suggest that a lower safety margin can be applied when using the proposed method.

Figure 8C,D, show scaled calculations that apply worst‐case SAR‐underestimation values as corrective safety margins to ensure SAR is never underestimated. For the initial position‐unaware calculations, this scaling emulates a safety model that includes all simulated positions. This correction limits the imaging performance across different positions to as low as 21% of the maximum due to SAR overestimation (indicated as a performance‐limiting factor in Figure 8C,D). That is, the worst‐case overestimation becomes 4.8‐fold with the safety margin in place. For the proposed position‐aware approach, worst‐case overestimation is less than 1.5‐fold with the safety margin, which ensures more than 68% of the maximum performance can be achieved across all cases, highlighting the performance gain enabled compared to the standard position‐unaware approach.

The Kolmogorov–Smirnov test established that the effect of subject motion on psSAR was significant (*D*(385) = 0.10, *p* = 0.035) while the difference between the ground‐truth and network‐estimated psSAR values was insignificant (*D*(385) = 0.03, *p* = 0.99), supporting our hypothesis.

## 4 Discussion

This study proposes to use deep learning to predict the effects of subject motion on local SAR distributions. Previous studies in the literature showed that subject motion can have a considerable effect on SAR calculations [13, 16]. Our investigations demonstrate that neural networks can estimate these motion‐induced changes, yielding local SAR distributions that are in close agreement with the actual ground‐truth. This approach can reduce motion‐induced SAR underestimation, thereby decreasing the corrective safety margins that are needed to ensure that peak local SAR is not underestimated. Because corrective safety margins increase SAR overestimation, and therefore restrict imaging performance, the proposed approach can lead to higher performance imaging.

We observed that anterior and posterior displacements caused much larger changes in SAR, in alignment with previous literature [13]. This larger increase in anterior–posterior direction is attributed to the head being naturally closer to the coil array in anterior and posterior directions due to head shape. As the head moves, the relative proximity to the coils increases more rapidly—for example, for a coil‐tissue distance of A20 mm and R40 mm, a 10 mm motion would reduce distance by 50% along anterior but only by 25% along rightward direction. Across positions, we found a monotonic increase in motion‐induced SAR error with increasing displacement from the initial position, similar to previous literature [13], with a single exception resulting from anterior–posterior displacements causing more rapid error increase.

Following a similar approach to previous literature [21], we trained separate networks for different motion types, which can then be cascaded to approximate actual patient position. This approach provides an efficient training solution compared to training a single network for all types of motion, which would require a much larger training dataset and training separate networks for each possible subject position, which would require too many networks and increase the total training cost.

Training the same network with a smaller dataset affected estimation performance, indicating that the training datasets in this study are not “over‐sized”. Consequently, the performance of the method can potentially be increased further with a larger training dataset.

Cascading networks trained for smaller displacements but with more data yielded better estimation performance compared to using networks trained for the exact amount of displacement but with less data. Interestingly, cascading the alternative P5 network trained with a smaller dataset also yielded better outcomes than the purpose‐trained P10 network. While this could again be attributed to the small difference between training dataset sizes, it could also indicate that networks trained on smaller displacements yielding small motion‐induced error might be more stable than networks trained on larger displacements that cause large motion‐induced error.

We investigated the estimation error floor and observed that each network adds around 1.5% estimation error. This error was deemed acceptable, being much smaller than the effect of motion on SAR. To reduce the number of cascades, multiple networks may be trained for each motion type (e.g., R2 and R5 for rightward) as proposed by Plumley [21]. This would also improve the approximation accuracy of actual subject motion; i.e., having L5, L2, R2, and R5 networks would allow cascading to all right–left positions with 1 mm intervals instead of 5 mm, enabling subject motion approximation with better than 0.5 mm accuracy, which is foreseen to be sufficient. Here we tested an example case where the networks were trained for a position 1 mm further away than the actual displacement and the estimations still reduced motion‐induced error considerably. This expands the utility of the proposed approach, as very precise estimation of organic motion is not crucial.

With multiple possible ways to cascade networks to the same result, we investigated the effect of cascading order. We observed that cascading the R5 networks earlier in the chain yielded marginally smaller nRMSE. Because anterior–posterior motion yielded larger errors and the training dataset for anterior–posterior motion was smaller, anterior–posterior estimations had larger estimation error. Introducing this larger estimation error earlier in the cascading chain likely led to a larger error at the final output. Nevertheless, the observed effect on estimation accuracy was small, indicating the robustness of the cascading approach when estimating changes in SAR.

Previous studies showed that subject motion can have a major impact on SAR estimations [13]—a behavior shown to be consistent across different body models [37]. Here, we observed similar motion‐induced SAR behavior, while numerical differences can be attributed to different body models used. Even in the absence of motion, Kopanoglu [34] showed that initial patient positioning affects SAR considerably. In practice, MR scanners use fixed safety models that comprise body models at all possible positions, which prevent underestimation, but are substantially over‐conservative and may therefore hamper imaging performance. Akin to Plumley's previous work [21] that used $B_{1}^{+}$ maps at the initial position to estimate $B_{1}^{+}$ maps at other positions, this work can transform Q‐matrices at the initial position to Q‐matrices at other subject positions, facilitating position‐adaptive safety models. It can be combined with any real‐time pulse design method [20, 34], which would also benefit from updated $B_{1}^{+}$ maps to adapt the excitation to motion as it happens [21]. This would reduce the need for over‐conservative, position‐wise, all‐inclusive safety models without risking SAR underestimation.

This method is compatible with and complementary to recent methods in the literature that focus on patient‐specific safety models [17, 18]. Because patient‐specific safety models are inherently position‐specific, their validity might be affected by motion until the models are updated after motion. This method can take those patient‐specific safety models and motion tracking information as input and provide position‐adaptive safety models. The envisioned method is sequence‐agnostic and agnostic to motion‐tracking methods; therefore, pertinent tools are not specified here.

For the implementation here, total computation time was not a constraint. Therefore, a sequential slice‐by‐slice estimation approach was adopted, with the estimation for a single slice taking around 16 ms. A further investigation might adopt a three‐dimensional estimation approach to estimate motion‐induced changes across slices together instead of sequentially to accelerate computation.

Q‐matrix and SAR distributions have rather smooth spatial variations, which means adjacent slices contain overlapping information. This can positively bias network‐estimation performance when training, validation and testing datasets have interleaved slices [19]. Therefore, we adopted the approach of splitting the datasets at a body model level, ensuring that the test dataset is completely unseen during training. In our limited investigations, we observed that the performance loss with the approach of splitting datasets at body model level was minor, and it is a more realistic approach as neural networks would be expected to perform well on unseen subjects in practice.

Despite the benefit of splitting datasets at the body model level in terms of testing reliability in unseen conditions, it also means that particularities of the anatomy used for testing might not be represented in the training dataset. Here, we tested the networks in a fourfold cross validation investigation across female pre‐teenager, female adult, male obese adult, and male elderly adult models. For smaller displacements, the networks yielded similar performance for all models, while the improvement provided by the networks was lower for the highest‐BMI model than the others for larger displacements. This is likely due to the large mismatch in BMI values across the training/validation (15.3 < BMI < 22.4), and testing (BMI: 36) datasets in this instance. In addition to all models having varying BMIs, they are also from different demographics (female child to elderly adult male), which may affect SAR calculations due to variations in tissue composition, and head size and geometry. For practical implementation, networks trained on more diverse datasets taking into consideration the effect of factors like anatomical variations (head shape and size), BMI, change in tissue characteristics with age, and presence of pathology or inflammation may yield more reliable and more generalizable performance. Another approach could be to train tailored networks for specific populations (e.g., low‐BMI vs. high‐BMI, pediatric vs. adult, healthy vs. certain pathology), which could make safety models more tailored to the population and reduce over‐estimation.

Finally, the proposed approach was evaluated using simulated data only, and its applicability to experimental data requires further validation. However, because U‐Nets operate on hierarchical image features at multiple spatial scales, and experimental data exhibit similar structural characteristics, we anticipate that the method will generalize to experimental settings.

## 5 Conclusion

This proof‐of‐principle study proposes a way to adapt safety calculations to patient motion as it happens, by estimating the effect of motion on SAR using deep learning. This can reduce the safety margins required to ensure adherence to safety limits. Despite its limitations, the proposed approach demonstrates a feasible alternative to using high‐performance but risky safety models that might underestimate SAR and all‐inclusive safety models that hamper imaging performance.

## Funding

This work was supported by the Engineering and Physical Sciences Research Council, [Wales Data Nation Accelerator Award] Engineering and Physical Sciences Research Council has grant number EP/T517951/1, Wellcome Trust (204824/Z/16/Z).

## Data Availability

The codes that support the findings of this study are available from the corresponding author upon reasonable request.

## Appendix A

For the *N*(*N*−1) two‐coil‐only Q‐matrix—ilSAR conversions, two combinations exist for each coil pair, where the coefficients are $\left(c_{m},c_{n}\right)$ and $\left(c_{m},\tilde{c_{n}}\right)$. The general inverse ilSAR—Q‐matrix mapping is given by:

(A1)

where the superscript * denotes complex conjugate.
